# In vivo effect of *Commiphora swynnertonii* ethanolic extracts on *Trypanosoma congolense* and selected immunological components in mice

**DOI:** 10.1186/s12906-017-1785-1

**Published:** 2017-05-23

**Authors:** Yakob P. Nagagi, Richard S. Silayo, Claudius Luziga, Eliningaya J. Kweka

**Affiliations:** 1 Department of Microbiology, Parasitology and Immunology, College of Veterinary and Medical Sciences, Sokoine University of Agriculture, P. O. Box 3019, Chuo Kikuu, Morogoro, Tanzania; 2 Tropical Pesticides Research Institute, Division of Livestock and Human Diseases Vector Control, P.O. Box 3024, Arusha, Tanzania; 3 Department of Anatomy, Histology and Cell Biology, Sokoine University of Agriculture, P. O. Box 3016, Chuo Kikuu, Morogoro, Tanzania; 4 Department of Medical Parasitology and Entomology, Catholic University of Health and Allied Sciences, P.O. Box 1464, Mwanza, Tanzania

**Keywords:** *Commiphora swynnertonii*, *Trypanosoma congolense*, Immunity

## Abstract

**Background:**

The search for alternative trypanocidal compounds which can be available at affordable price is of paramount importance for control of trypanosomosis in human and animals. The current study evaluates the in vivo activity of ethanolic stem bark extracts on *Trypanosoma congolense* and selected immunological components in an inbred Swiss albino mouse model.

**Methods:**

Groups of mice infected with *T. congolense* were treated with the stem bark extracts at a rate of 1000 mg/kg, 1500 mg/kg, and 2000 mg/kg, twice a day in one set and thrice a day in another setting for three days consecutively. Negative (infected and untreated) and positive (infected treated with diminazene diaceturate at 3.5 mg/kg) control groups were used. Levels of parasitaemia were monitored daily for the first 10 days and thereafter 2–3 times per week to the end of experiment. In the other setting, uninfected mice, randomized in groups were treated with the extract but categorized as: thorough mixed extract (TME) and supernatant extract (SE) each at 500 mg/kg and 1500 mg/kg, in 8 hourly intervals respectively for three days consecutively. Control group was administered with phosphate buffered saline with glucose at 0.1 ml/10 g in a similar manner as for the extract. Whole blood and spleen were taken 24 h after the last treatment for hematological and histopathological analysis.

**Results:**

The groups that received the extracts at 8 hourly intervals drastically reduced the parasitaemia. The higher dose of SE significantly reduced the percentage of lymphocytes (*P* < 0.05). Both high and low dose of TME significantly reduced lymphocytes percent (*P* < 0.05) while percent of neutrophils and monocytes increased significantly (*P* < 0.05). Histopathological changes of the spleen in the mice treated with higher concentrations of the extract of *C. swynnertonii* were suggestive of lymphocytes toxicity.

**Conclusion:**

The current study has provided evidence that, in vivo trypanocidal activity of ethanolic bark extracts of *C. swynnertonii* is probably affected by its negative effect on humoral mediated immune response. Further studies are recommended to determine its potential as an alternative source of lead compounds for trypanocidal drug discovery.

## Background

African trypanosomosis is one of the many constraints that hinders Africa’s struggle against poverty through livestock keeping. It is an important infection that greatly affects humans and livestock in 37 African countries of which 21 are the world’s poorest countries [1]. The important species and subspecies, *T. brucei gambiense* and *T. b. rhodesiense* are known to cause Human African trypanosomiasis (HAT) [2] and *T. B. brucei*, *T. congolense, T. vivax* and *T. simiae* which are infectious to animals are known to cause Animal African trypanosomiasis (AAT) [3]. Despite the major reduction in the number of new cases in the recent past, HAT remains an important public health problem in the affected countries [4]. On the other hand, AAT is one of the major livestock production constraints leading to 34% of livestock keepers to subsist on less than 1.24 USD per day [5]. Therefore, control of AAT is imperative due to its massive impact on the livelihood of rural communities [6]. However, the current disease control tools which rely on extensive use of chemotherapeutic agents is outdated and blunted by overuse practices [7], leading to failure and or reduced efficacy against emergent trypanosomes resistant strains [7, 8]. The problem is compounded by lack of interest of industries to develop new drugs due to high costs of such endeavor versus expected return from poor affected African countries [9]. Nevertheless, there is a need for increased scientific interest in searching for new anti-trypanosome molecules that the industry could take up for further development into new trypanocidal drugs. One such source of candidate molecules are plants.

Plants remain to be essential in healthcare with earliest records dating back from around 2600 BCE documenting the uses of approximately 1000 plant-derived substances in Mesopotamia [10]. Currently, the advancement in scientific techniques has lead to isolation and identification of thousands of phytochemicals from plants. Many of these phytochemicals are leading sources for developing chemotherapeutic drugs against a number of diseases (infectious and non-infectious) [11–13]. *Commiphora swynnertonii* which belongs to the family Burseraceae is the most famous medicinal plant species in northern Tanzania. It has a number of medicinal purposes such as: treatment of sexually transmitted diseases, ulcers and wounds (cut and burn wounds), recalcitrant ulcers, abscesses, swelling of legs, chesty cough and scabies [14]. Its resinous exudates are used for treatment of worm infestation, dental caries, cleansing bladder and control of parasites such as ticks, lice, bed bugs and mange mites [14]. Indeed, previous studies have provided a number of information about *C. swynnertonii* to support its traditional medicinal use [15–20].

The ethanolic stem bark extract of *C. swynnertonii* has recently been shown to possess trypanocidal activity in vitro [21]. However, extracts effective in vitro are not necessarily active also in vivo [22]. Among the contributing factors include (i) active compounds in the extracts may be metabolized too quickly to a less active or inactive form, (ii) the efficacy of the extracts may rely on not disrupting the immune system or its ability to activate the defense mechanism in a way that facilitate clearance of the infectious agent.

The aim of the present study was to determine the in vivo activity of extracts of *Commiphora swynnertonii* Burtt in an inbred Swiss albino mouse model infected with an isolate of *Trypanosoma congolense*. In addition, the study, reports the effect of the extracts on circulating white blood cells and spleen in infected mice. Its implication on the antitrypanosomal efficacy is discussed.

## Methods

### Plant materials

Plant stem bark pieces were collected from Kitwai A village (04°05′42.00″S and 36°33′34.42″E), in Simanjiro district in Manyara region in Northern Tanzania. The plant specimen was submitted to National Herbarium of Tanzania, Tropical Pesticides Research Institute, Arusha (Specimen voucher Number CS-01). Confirmation of Plant species was done by Emmanuel Mboya, a plant taxonomist. The collected plant materials were transported to Sokoine University of Agriculture for preparation, extraction, in vitro and in vivo testing.

### Plant extract preparation

The stem bark was peeled off and dried under shade for 4 weeks. The dried barks were ground to fine powder using laboratory mill (Christy Hunt Engineering Ltd., England) and stored in an airtight bag in a cool dry room until used. In one hand, five hundred grams (500 g) of the ground stem bark was weighed and soaked in 1000 ml of 99.9% ethanol in a conical flask sealed with aluminium foil and left for 72 h in a dark place with occasional stirring. On the other hand, fifty grams (50 g) of ground stem bark was weighed and soaked in 100 ml of 99.8% methanol in a similar manner as for the ethanol. Each mixture, was filtered using a piece of cotton wool in a funnel into a conical flask and then using Whatmann® filter paper No. 1. The obtained filtrates were put in a separate beaker and concentrated by evaporation under ceiling fan at room temperature. The resulting crude extracts were then stored at 4 °C in airtight bottles until used.

Sixteen (16) grams of stem bark extract was weighed into a bijou bottle and diluted with 80 mls of phosphate buffered saline with glucose (PBSG) (pH, 8.0) to make 200 mg/ml stock solution. Three other extract concentrations (150 mg/ml, 100 mg/ml and 50 mg/ml) were prepared from stock solution by serial dilutions using PBSG. The remaining stock solutions were stored in a refrigerator at 4 °C until required.

### Experimental animals

Male, random-bred, Swiss albino mice 2½ months old, with mean weight (mean ± SD) 29.71 ± 4.8 g were used in carrying out the studies. The mice were obtained at the Small Animal Unit of the College of Veterinary and Medical Sciences, Sokoine University of Agriculture.

### Housing and husbandry

Handling of animals was done in accordance to OECD guidelines [23]. The mice were kept in plastic cages 275 × 160 × 130 mm, five mice per group with wood shavings as bedding and identified with picric acid markings. They were fed broiler mash (finisher) from local feed manufacturer and boiled tap water was provided adequately. They were given four days to acclimatize to the housing facility prior to treatment. The mice that participated on the in vivo activity of extract of *C. swynnertonii* on *T. congolense* study were continually provided with feeds and water adequately even after termination of the experiment. All sections of this report comply with ARRIVE guidelines for reporting animal research [24].

### Trypanosome stock

The trypanosome stock used in this study was a stock of putative drug sensitive strain *Trypanosoma congolense* originally isolated from Mikese, Morogoro and maintained by serial passage in Swiss albino mice at the Small Animal Unit of the College of Veterinary and Medical Sciences, Sokoine University of Agriculture.

### Monitoring of Parasitaemia

Parasitaemia in mice was monitored by microscopic examination of wet smear from mouse tail blood using the method described by Herbert and Lumsden [25]. This involved counting of parasites per field in pure blood. Logarithm values of these counts were obtained by matching with table of Herbert and Lumsden [25] and or converted to antilog to provide absolute number of trypanosomes per milliliter of blood.

### Acute toxicity study

A study on acute toxicity was conducted according to OECD guideline for testing of chemicals using mice at a dose of 2000 mg/kg [23]. Five female Swiss albino mice received orally 2000 mg/kg of the ethanolic stem bark extract. After which they were continuously observed for 30 min then periodically during the 24 h with special attention given for the first four hrs and thereafter daily for a total of 14 days. This study indicated that there were no deaths and no visible signs of acute toxicity in the mice treated at the dose tested (2000 mg/kg) during the 14 days observation period.

### In vivo activity of the extract on *Trypanosoma congolense*

**Fig. 1:**
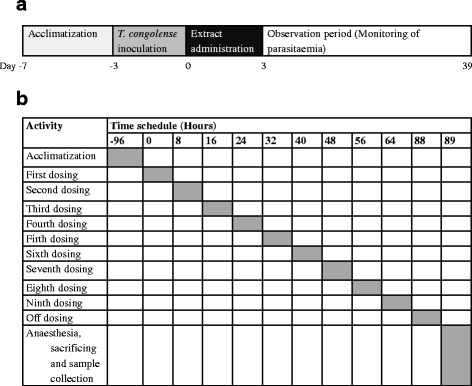
Experimental timeline, **a** Timeline trend of major events for the experiment that was assessing the in vivo activity of *C. swynnertonii* extract on the parasitaemia of *T. congolense* in mice. **b** Timeline schedule extract administration for the experiment that was assessing the effect of *C. swynnertonii* extract on some immunological components in mice

A sum of 40 clinically health mice were inoculated intraperitoneally with approximately 3 × 10^5^ trypanosomes. The mice had parasitaemia of approximately 2.5 × 10^5^ trypanosomes per milliliter of blood on the third day. They were then assigned with numbers 1–40, and randomized using a RAND function in the Microsoft excel. This resulted into eight experimental groups (G1 – G8) of five mice each. The number of experimental groups and that of mice per group were derived based on standardized protocol recommended by Eisler and colleagues [26] with some modifications. The mice in G1 – G6 were administered with ethanolic extract of stem bark of *C. swynnertonii*. Those in G7 were treated as negative control while in G8 as positive control. A time line diagram for this experiment is shown in Fig. 1, a.

The dose and treatment regime was selected based on acute toxicity study and previous trial experiments. The extracts were thoroughly mixed before administration. The mice in G6, G4, G3 received the extract at a dose of 1000 mg/kg, 1500 mg/kg, 2000 mg/kg respectively, at 12 hourly intervals for three days consecutively. The mice in G5, G1, G2 received the extract at: 1000 mg/kg, 1500 mg/kg, 2000 mg/kg, at 8 hourly intervals for three days consecutively. The mice in G7 did not receive any treatment, while those in G8 were treated with (diminazine diaceturate (Veriben®, Ceva Santé Animale, France) at a dose of 3.5 mg/kg intraperitoneally as a single injection. Monitoring of parasitaemia was done daily for the first 10 days and thereafter intermittently 2–3 days per week until the 39^th^ day post inoculation. For any mouse that was found dead, was examined for postmortem changes, the spleen and any organ(s) with noticeable change were taken in 10% neutral buffered formalin for histopathology.

### Analysis of the extract

Two hundred milligrams (200 mg) of methanolic extract was weighed and diluted with 2 mls of methanol (99.8%), the resulting solution was used for chemical composition analysis using gas chromatograph mass spectrometry (GC-MS), Agilent Technologies. The GC-MS conditions: helium as a carrier gas, 1.2 ml/min flow rate, 30 m column length, 0.25 mm internal diameter, 0.25 mm film thickness, ion source temperature 230 °C, injection mode-autoinjector with split-splitless, mass spectrometry detector inlet temperature at 250 °C, temperature of m/s quandrapole at 150 °C and pressure vacuum at 4.85 × 10^−5^ psi.

### Effect of extract on selected immunological components

To assess the effect of the extract on selected immunological components, another experiment was carried out. Twenty five clinically healthy mice were assigned numbers 1–25 and randomized as above into five groups (G9 – G13) of five mice each. The mice in G9 – G12 were administered with the ethanolic stem bark extract of *C. swynnertonii* while mice in G13 received PBSG only (control). A time line diagram for this experiment is shown in Fig. 1, b.

Mice in G9 and G12 were administered with 500 mg/kg, and 1500 mg/kg respectively of thoroughly mixed extract (TME). Mice in G10 and G11 were administered with 500 mg/kg, 1500 mg/kg of supernatant extract (SE) whereas G13 received 0.1 ml/10 g of PBSG only. The treatment was done at 8 hourly intervals for three days consecutively. Twenty four hours after the last treatment, the mice were weighed, anaesthetized and sacrificed. Immediately, blood from each mouse was collected in EDTA vacutainer tubes in ice packs, spleen was excised and placed in bijou bottle containing 10% neutral buffered formalin. Later, total and differential white blood cell count was determined using an automatic haematological analyzer.

### Hematoxylin and eosin staining

Organs with noticeable postmortem changes and the spleen were prepared for histopathology assessment. Three replicates from the liver, kidney and spleen sections of 5 μm per treatment were cut and processed by rapid manual tissue processing as described in Culling [27]. The processed sections were stained with hematoxylin and eosin (H & E) for histopathological observations.

### Immunohistochemistry

#### Tissue preparation

Again, spleen tissues from mice treated with TME and SE of C. *swynnertonii* at a dose of 1500 mg/kg at 8 hourly intervals for three days consecutively were dissected. Spleen tissues from mice treated with 0.1 ml/10 g of PBSG only were also included. They were post-fixed with 4% paraformaldehyde (PFA; Sigma-Aldrich, St. Louis, MO) in 0.1 phosphate buffer (PB; pH 7.4) for 2 hrs at 4 °C before processing to paraffin wax and sectioning.

#### Immunostaining procedure

The procedure for immunostaining was done as described by Luziga and colleagues [28] with some modifications. The sections were deparaffinized in xylene then rehydrated through a descending ethanol series to phosphate-buffered saline (PBS). To inhibit endogenous peroxidase activity, sections were immersed in a 0.3% *v*/v hydrogen peroxide in distilled water for 30 min at room temperature followed by washing (3 × 5 min) in 0.01 M PBS, pH 7.4. Sections were then incubated with 10% normal goat serum in PBS for 1 h at room temperature to block non-specific binding. To detect single stranded DNA, a marker for apoptotic cells, sections were incubated with polyclonal rabbit anti-ssDNA antibody (Immuno-Biological Laboratories Co., Ltd., Code No 18731) at a dilution of 2 μg/ml for 24 h in a dark, humid chamber at 4 °C. For the negative control, PBS was applied in place of primary antibody. Sections were washed (3 × 10 min) in PBS, before incubation with streptavidin-peroxidase conjugate for 30 min at room temperature. Visualization of binding sites was accomplished by incubating the sections for 3–5 min with a medium containing 0.05% 3,3-diaminobenzidine tetra-hydrochloride in 0.015% hydrogen peroxide and 0.01 PBS, pH 7.2 for 1–3 min at room temperature. The sections were counterstained with Mayer’s hematoxylin for 30 s, rinsed for 15 min in running tap water and then dehydrated through a graded alcohol series, cleared and mounted in DPX. Immunolabeling was analyzed using a Olympus BH-2 microscope fitted with Olympus camera. For immunofluorescence labeling, the initial steps in processing tissues remained the same as for the streptavidin-peroxidase method. However, instead of incubating with streptavidin-peroxidase, the sections were incubated for 1 h at room temperature with Alexa Fluor® 488-conjugated goat anti-rabbit IgG (FITC) at a dilution of 1:100 (abcam). At the end of incubation, the sections were washed (3 x 5 min) in PBS and mounted, followed by visualization of the binding sites using fluorescence microscope.

### Experimental outcomes

This study provides the in vivo activity of *C. swynnertonii* extract on *T. congolense* parasitaemia in a mouse model. The study also report on the effect of the extract on the white blood cells and the spleen as part of the components involved in the host immune response against pathogens.

### Statistical methods

Data on levels of parasitaemia, total and differential white blood cell counts were presented as mean ± standard deviation. Statistical analysis was done using one way analysis of variance (One way ANOVA) in the statistical package for social science (SPSS) version 16 (Chicago, SPSS Inc., USA). Excel program was used to determine the trend in the levels of parasitaemia and total white blood cell counts in the respective treated groups.

## Results

### In vivo activity of the extract on *T. congolense*

**Fig. 2:**
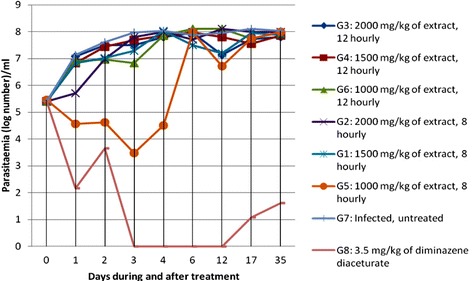
Trend of the mean parasitaemia of *T. congolense* during and after treatment of extract of *C. swynnertonii*

The effect of the extract of *C. swynnertonii* on the parasitaemia of *T. congolense* during and after treatment is shown in Fig. 2. There were fluctuations in the parasitaemia of *T. congolense* in treated mice. Mice which received the extract at 8 hourly intervals had a moderately lower parasitaemia during therapy than those at 12 hourly intervals. The group, G5, which received the extracts at 8 hourly intervals had initially drastically reduced the parasitaemia. However, G2 (2000 mg/kg) was the only group that showed a significantly ﻿(﻿*﻿P*﻿﻿ < 0.05) lower parasitaemia after the first day of therapy compared to negative control (Table 1). There was a significant ﻿﻿(*P* < 0.05) ﻿﻿reduction of parasitaemia in G5 (1000 mg/kg) on the 2^nd^, 3^rd^ and 4^th^ day of therapy whereby some of the mice completely cleared the parasite as per observations by wet smear. Parasitaemia in G8 (diminazene diaceturate at 3.5 mg/kg) was significantly (*P* < 0.05) lower than the negative control until the 2^nd^ day post treatment. Thereafter, parasitaemia was not observed in blood by wet smear until the 17^th^ day when few mice had parasitaemia.

**Table 1:** Effect of extract of *C. swynnertonii* on parasitaemia in mice infected with *T. congolense*

		Parasitaemia level (logarithm number)/ml			
Days	G2	G1	G5	G7	G8
0	5.4 ± 0.00	5.4 ± 0.00	5.46 ± 0.134	5.4 ± 0.00	5.4 ± 0.00
1	5.7 ± 0.3^*^	6.84 ± 0.39	4.56 ± 2.56	7.14 ± 0.65	2.16 ± 2.96^*^
2	6.98 ± 0.38	7.02 ± 0.94	4.62 ± 2.60^*^	7.62 ± 0.34	3.66 ± 3.45^*^
3	7.8 ± 0.3	7.28 ± 1.08	3.48 ± 3.19^*^	7.98 ± 0.34	0.00 ± 0.00^*^
4	8 ± 0.35	8.03 ± 0.29	4.5 ± 2.55^*^	8.04 ± 0.25	0.00 ± 0.00^*^
6	7.7 ± 0.62	7.5 ± 0.42	7.98 ± 0.16	7.88 ± 0.29	0.00 ± 0.00^*^
12	8.1 ± 0.3	7.2 ± 0.55	6.72 ± 1.09	7.95 ± 0.17	0.00 ± 0.00^*^
17	8 ± 0.17	7.95 ± 0.17	7.74 ± 0.13	7.95 ± 0.30	2.16 ± 2.96^*^
35	8 ± 0.00	7.88 ± 0.29	8 ± 0.15	8.03 ± 0.15	3.18 ± 4.36

Values are mean ± STDEV; Day 0: the day treatment started; STDEV: standard deviation; G2, G1, G5: the groups that received ethanolic stem bark extract of *C. swynnertonii* at rate of 2000 mg/kg, 1500 mg/kg, 1000 mg/kg respectively, 8 hourly for 3 days; G7: infected-untreated group; G8: group that received diminazene diaceturate at rate of 3.5 mg/kg; Superscript “*”indicate significance at *P* < 0.05 compared to untreated group

## Effect of extract on selected immunological components

### Effect of extracts on white blood cells

**Fig. 3:**
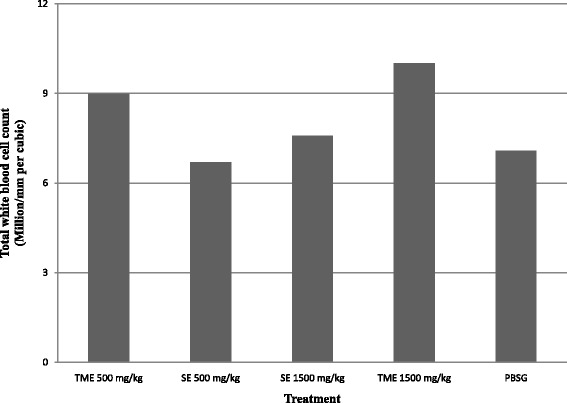
Trend in total white blood cell counts in extract treated groups

There was a slight elevation in the levels of the total white blood cell count (WBC) in the mice treated with the extract save for the group that received 500 mg/kg of SE (Fig. 3). The effects of the ethanolic stem bark extract of *C. swynnertonii* on differential white blood cell count in mice is shown in Table 2. The total WBC in mice treated with extracts did not vary significantly (*P* > 0.05) with the control (PBSG only). However, the percentage of lymphocytes decreased significantly in G9, G11 (*P* < 0.05) and G12 (*P* < 0.01) while that of neutrophils increased significantly in G9 (*P* < 0.05) and G12 (*P* < 0.01). Similarly, the percentage of monocytes was significantly﻿ ﻿(*P* < 0.05) higher in G9 and G12.

**Table 2:** Effect of ethanolic stem bark extract of *C. swynnertonii* on differential white blood cells in mice

White blood cells (%)	Experimental groups				
	G9 (4)	G10 (5)	G11 (5)	G12 (3)	G13 (5)
Lymphocytes	66.7 ± 12.99*	77.08 ± 6.90	62.70 ± 21.09*	65.37 ± 9.52**	85.32 ± 5.67
Neutrophils	21.08 ± 7.0*	15.96 ± 5.23	20.96 ± 9.35	23.07 ± 4.69**	10.9 ± 3.64
Monocytes	9.23 ± 4.61*	2.94 ± 2.40	6.88 ± 9.18	7.5 ± 4.19*	2.68 ± 0.60
Eosinophils	2.75 ± 1.98	3.74 ± 2.10	9.16 ± 10.85	3.77 ± 1.18	1.02 ± 2.28
Basophils	0.25 ± 0.26	0.28 ± 0.19	0.3 ± 0.21	0.3 ± 0.26	0.08 ± 0.18

Values are mean ± STDEV; STDEV: standard deviation; G9, G12: groups treated with TME at rate of 500 mg/kg, 1500 mg/kg respectively; G10, G11: groups treated with SE at rate of 500 mg/kg, 1500 mg/kg respectively; TME and SE stands for thorough mixed extract and supernatant extract of ethanolic stem bark of *C. swynnertonii*; G13: group treated with PBSG at the rate of 0.1 ml/10 g. The *stand for significance with respect to negative control whereas *: *P* < 0.05, **: *P* < 0.01. The number of mice from which the data were obtained is shown in the brackets

## Effect of extracts on spleen and other organs

### Macroscopic changes

The mice that were found dead had no noticeable macroscopic change in the heart, lungs, spleen and kidney. The liver had patches of brownish appearance on its surface while the intestines took the color of the extract which is greenish yellow appearance.

### Histopathology (H & E)

**Fig. 4:**
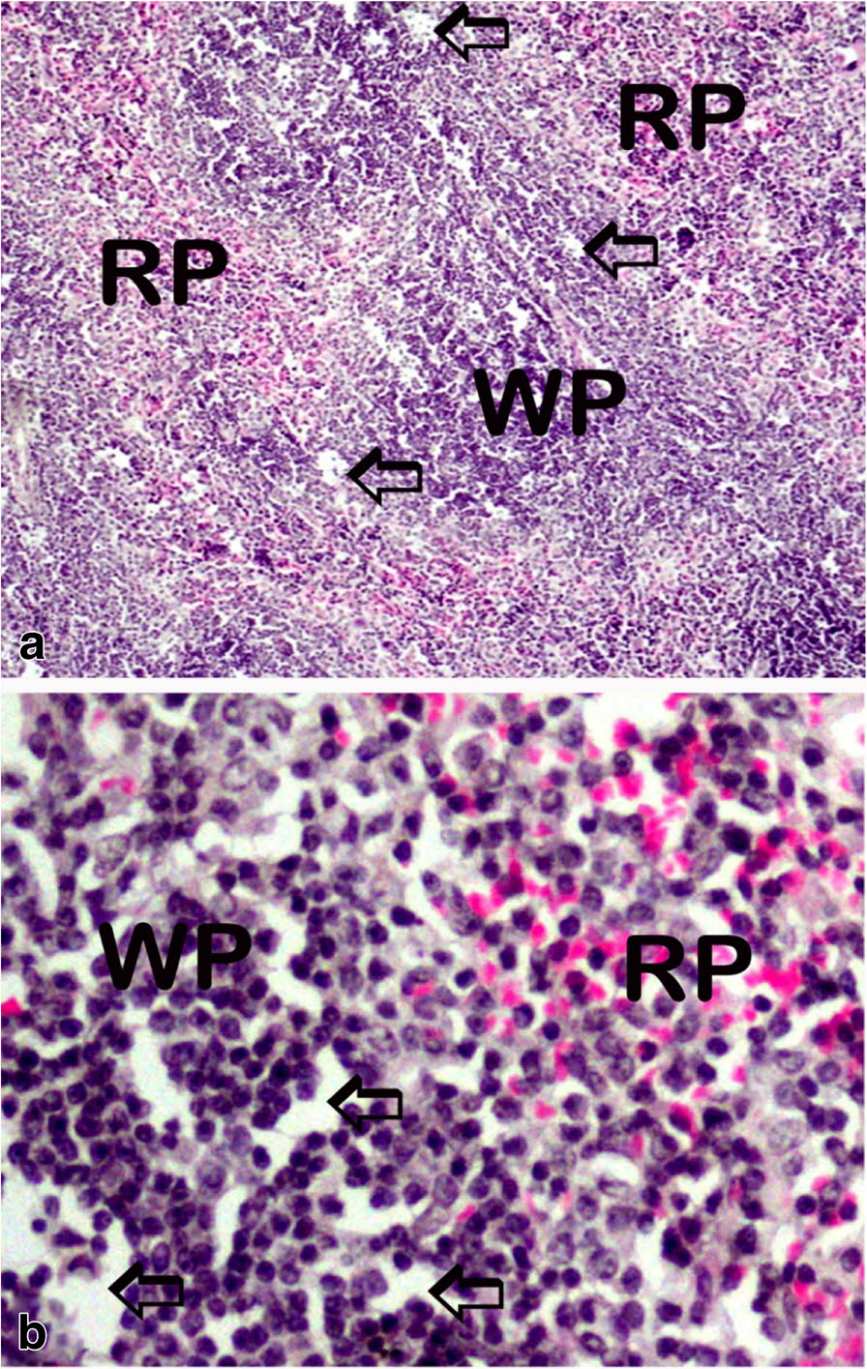
Histological sections of spleen from the mice in G11 treated with SE of *C. swynnertonii* (SE, 1500 mg/kg), 8 hourly intervals for three days consecutively. There are *white* foci of variable sizes in the *white* and *red pulp* (*open arrows*) in (**a**). Pyknosis of lymphocytes in some of the *white* foci is evident in (**b**). Magnifications, (**a**) 100 x; (**b**) 400 x

**Fig. 5:**
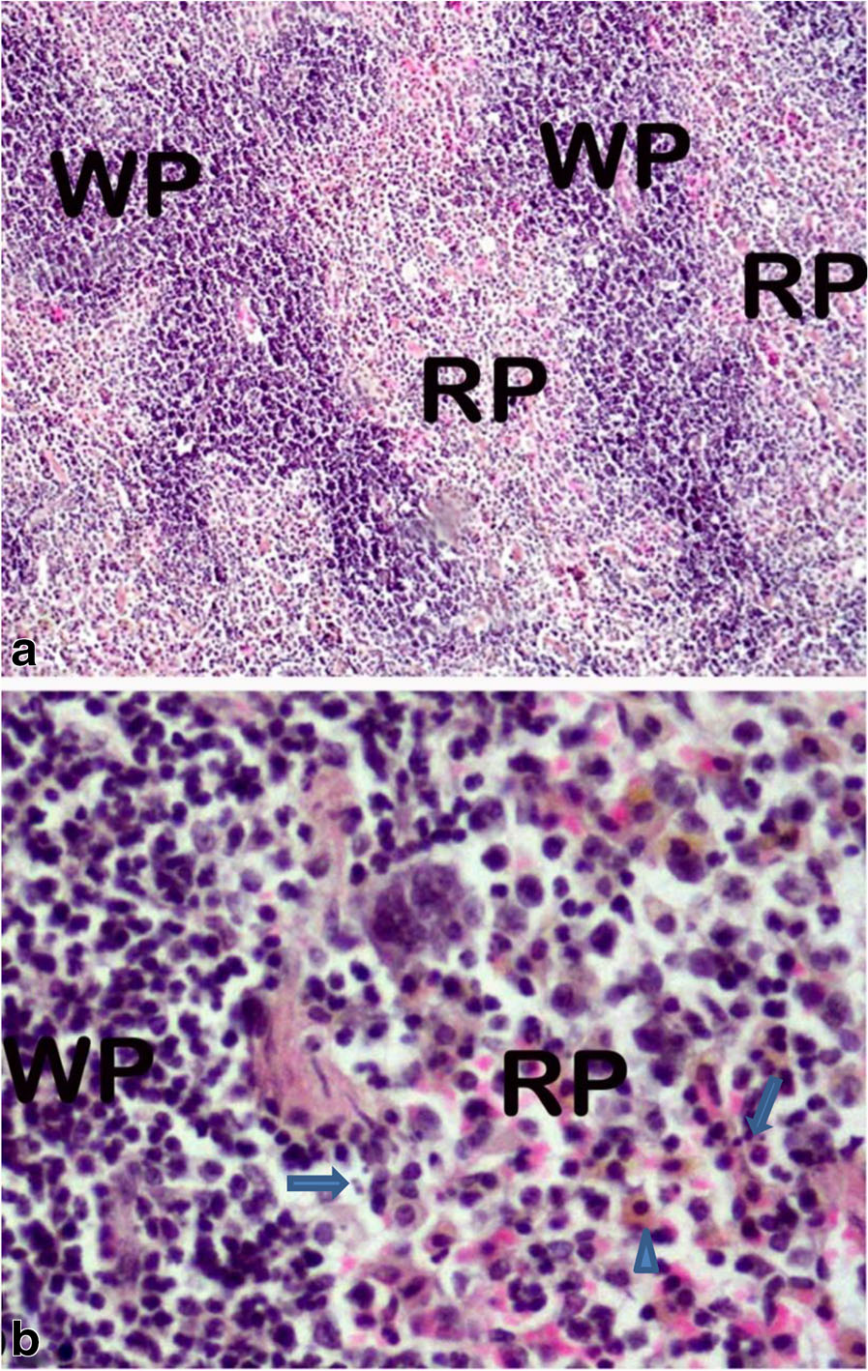
Histological sections of spleen from the mice in G12 treated with TME of *C. swynnertonii* (TME, 1500 mg/kg), 8 hourly intervals for three days consecutively. In (**a**), the* white* and *red pulps* are markedly reduced in size; the PALS has normal cellularity, lymphoid follicles and marginal zone are depleted. There is moderate apoptosis with karyorrhexis (*arrows*) and pyknosis of lymphocytes in (**b**). Magnifications, (**a**) 100 x; (**b**) 400 x

**Fig. 6:**
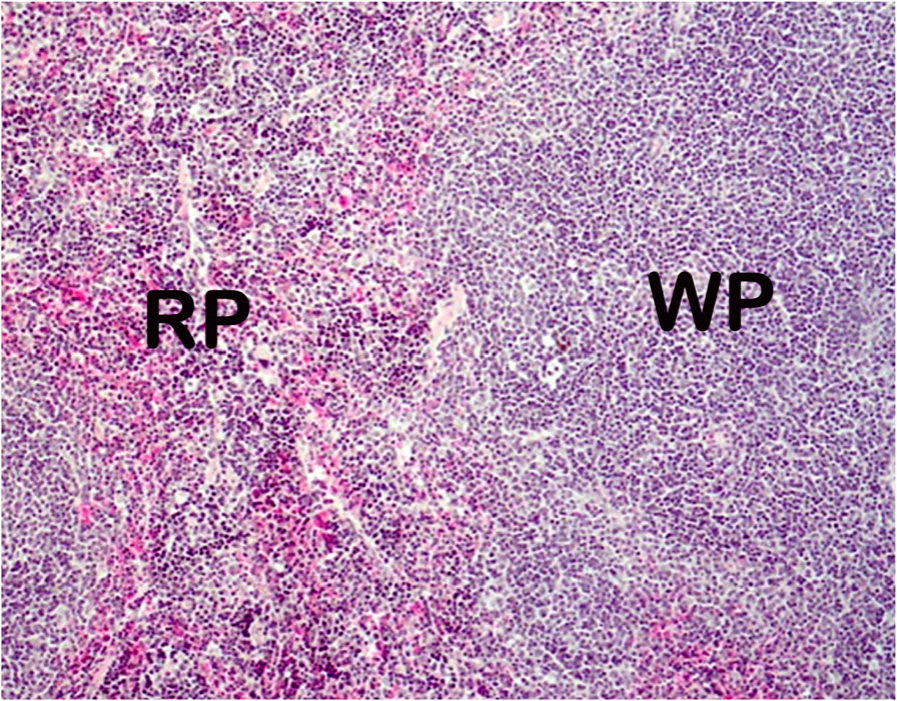
Histological sections of spleen from the mice treated with PBSG at the rate of 0.1 ml/10 g as the extract at 8 hourly for three days consecutive for comparison with the mice treated with extracts of *C. swynnertonii*. The *red* and *white pulps* have a normal size and cellularity. In the red pulp there are discrete areas of red indicative of congestion. Magnifications, 100 x

The various changes on the histological sections of the spleen from the mice that were treated with different concentrations of the extracts of *C. swynnertonii* are summarized in Table 3. The main distinguishing features were observed in spleen from the mice treated with a higher dose of both SE (G11) and TME (G12). There was a presence of white foci of variable sizes in the white pulp which extended into the red pulp in G11 (Fig. 4, a). At higher magnifications apoptosis characterized with pyknosis of lymphocytes in some of the foci was evident (Fig. 4, b). In G12, a marked reduction in the size of the white and red pulp, the periarteriolar lymphoid sheath (PALS) had normal cellularity, lymphoid follicles and marginal zones were depleted (Fig. 5, a). At higher magnifications, there was moderate apoptosis with karyorrhexis and pyknosis of lymphocytes (Fig. 5, b). The spleen section from the control group had normal size and cellularity in the white and red pulp (Fig. 6).

**Table 3:** Effect of *C. swynnertonii* ethanolic stem bark extract on spleen

Treatments	White pulp			Red pulp
	Perioarteriolar lymphoid sheath (PALS)	Lymphoid follicles	Marginal zone	
PBSG (0.1 ml/10 g)	Normal	Normal	Normal	Congestion
TME, 500 mg/kg	No change	No change	No change	Congestion
SE, 500 mg/kg	No change	No change	No change	Congestion
TME, 1500 mg/kg	No change	Marked reduction in cellularity and size, Apoptosis, pyknosis of lymphocytes and tingible body macrophage	Depleted	Cellularity and size severely reduced
SE, 1500 mg/kg	Apoptosis, white foci of variable sizes	-Apoptosis, -white foci of variable sizes, pyknosis of lymphocytes is evident in some of the foci. -Severely reduced in size	No change	Apoptosis, white foci of variable sizes
Infected, treated with 1500 mg/kg of extract but died on 3rd day	Apoptosis and white foci of variable size	-Apoptosis characterized by white foci and pyknosis of lymphocytes	A moderate widening	Congestion
Infected, treated with 2000 mg/kg of extract but died on 2nd day	No change	-Moderate apoptosis characterized with karyorrhexis of lymphocytes -Increased in size	A moderate widening	No change
Infected but not treated, died on 9th day post inoculation	No change	Severely reduced	No change	Increased cellularity and size

**Fig. 7:**
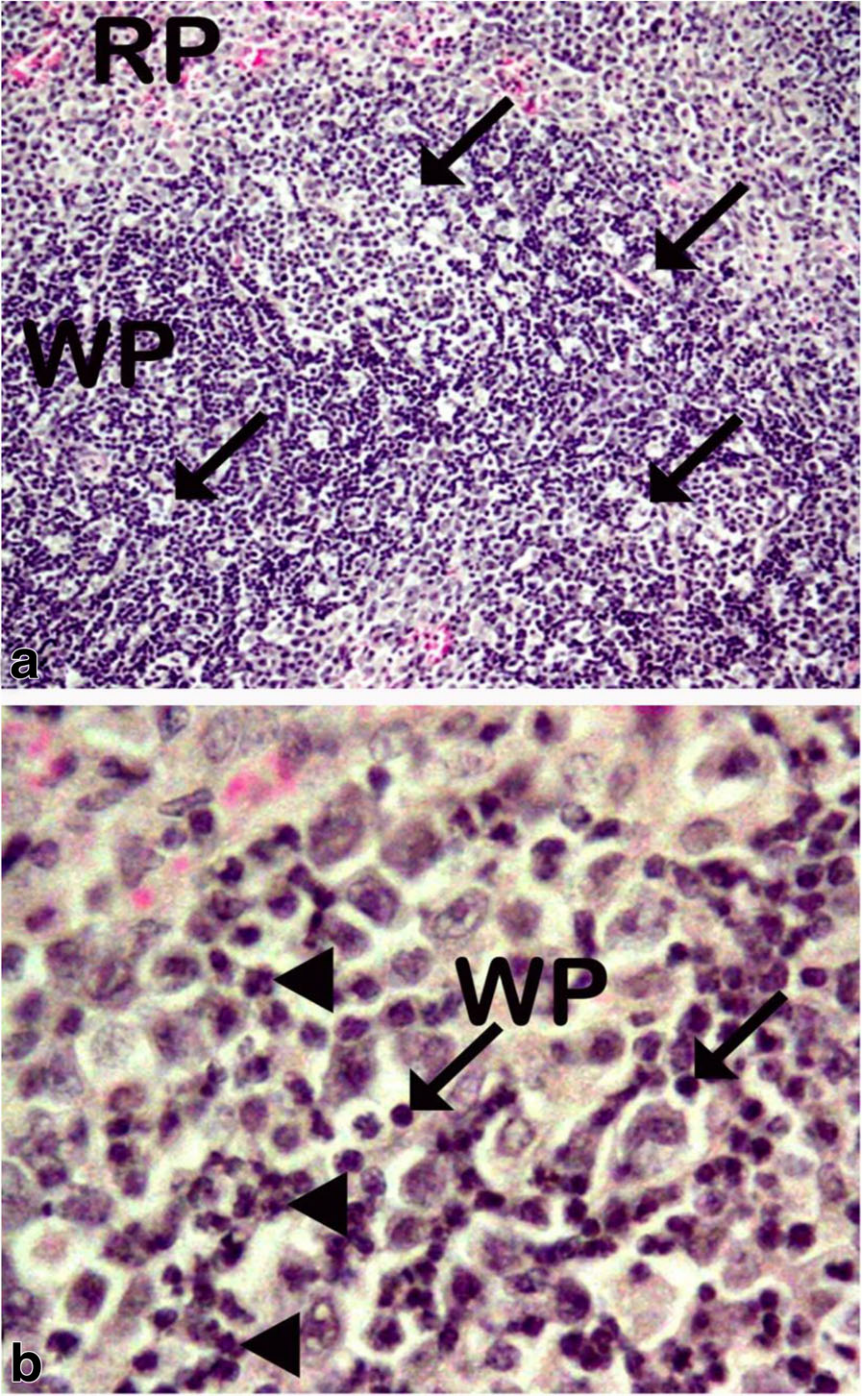
Histological sections of the spleen from mice in G1 infected with *T. congolense* and treated with 1500 mg/kg of *C. swynnertonii* extracts, 8 hourly intervals but died on third day of treatment. There are white foci of variable sizes in the white pulp (*black arrows*) in (**a**). Pyknosis (*black* arrows) and karyorrhexis (*arrow head*) of lymphocytes are evident in (**b**). Magnifications, (**a**) 100 x; (**b**) 1000 x

**Fig. 8:**
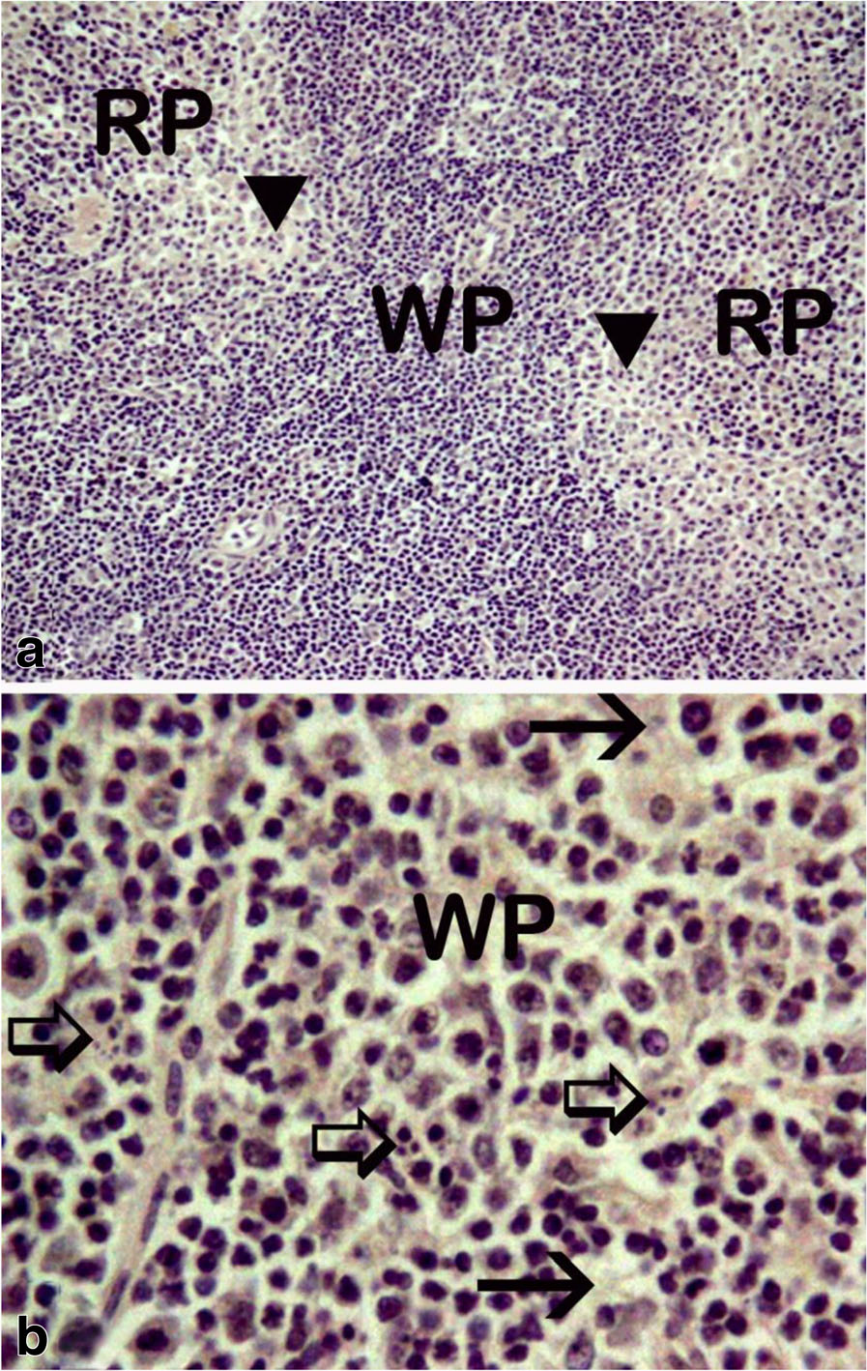
Histological sections of the spleen from mice in G2 infected with *T. congolense* and treated with 2000 mg/kg of *C. swynnertonii* extracts, 8 hourly intervals but died on 2nd day of treatment. There is expansion of the lymphoid follicles and widening of marginal zone (arrow heads) in (**a**). Apoptosis characterized with pyknosis (*black* arrows) and karyorrhexis (open arrows) of lymphocytes in (**b**). Magnifications, (**a**) 100 x: (**b**) 1000 x

**Fig. 9:**
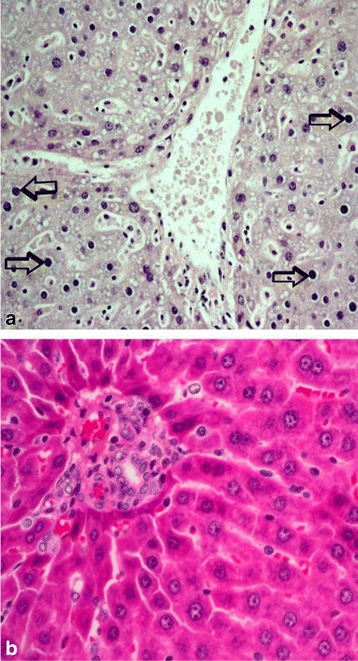
Histological section of the liver from mice that received 2000 mg/kg, 8 hourly intervals but died on 2nd day of treatment. There is a marked cytoplasmic vacuolation of hepatocytes, some pyknotic hepatocytes (*black* arrows) and Küpffer cell hyperplasia in (**a**). Section (**b**) is for comparison, has normal hepatocytes and cellularity. Magnifications, (**a** & **b**) 400 x

**Fig. 10:**
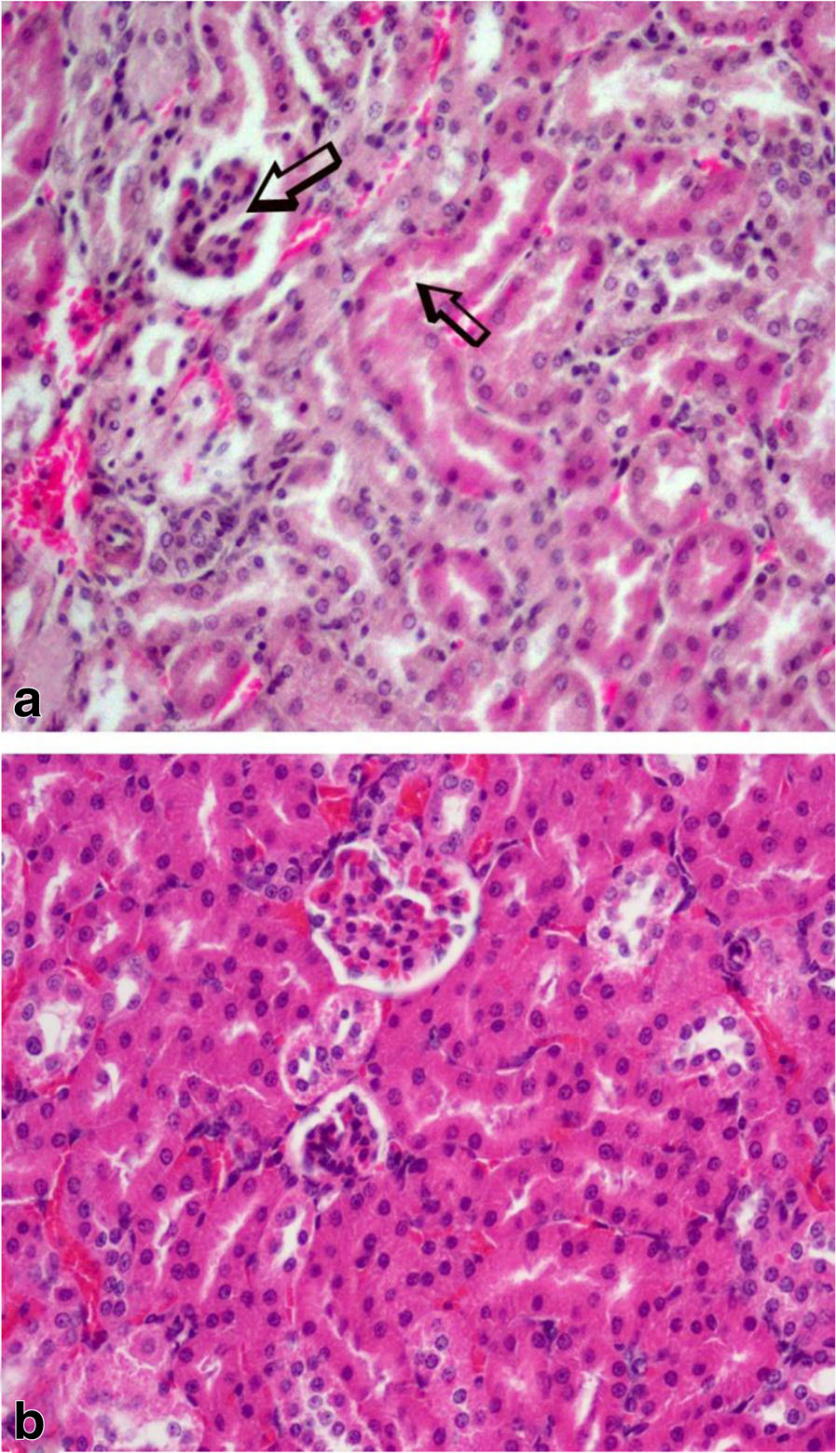
Histological section of the kidney from mice that received 2000 mg/kg, 8 hourly intervals but died on 2nd day of treatment. There is a mild hydropic degeneration of cortical-tubular epithelium and glomerulus in (**a**). Section (**b**) is from normal kidney for comparison. Magnifications, (**a** & **b**) 400 x

The changes observed from the spleen section of the mouse that received 1500 mg/kg of extract but died on the third day of therapy had necrotic foci of variable sizes in the white pulp and a widening of the marginal zone (Fig. 7, a). In addition, apoptosis characterized by pyknosis of lymphocytes was observed (Fig. 7, b). The spleen section from the mouse that received 2000 mg/kg, 8 hourly intervals but died on second day of treatment showed widening of lymphoid follicles and marginal zone (Fig. 8, a). At higher magnifications apoptosis characterized by karyorrhexis of lymphocytes were evident (Fig. 8, b). Also, the liver showed a marked cytoplasmic vacuolations of hepatocytes, some pyknotic hepatocytes and Küpffer cell hyperplasia (Fig. 9, a). The kidney had a mild hydropic degeneration of cortical-tubular epithelium and glomerulus (Fig. 10, a).

### Immunohistochemical findings

**Fig. 11:**
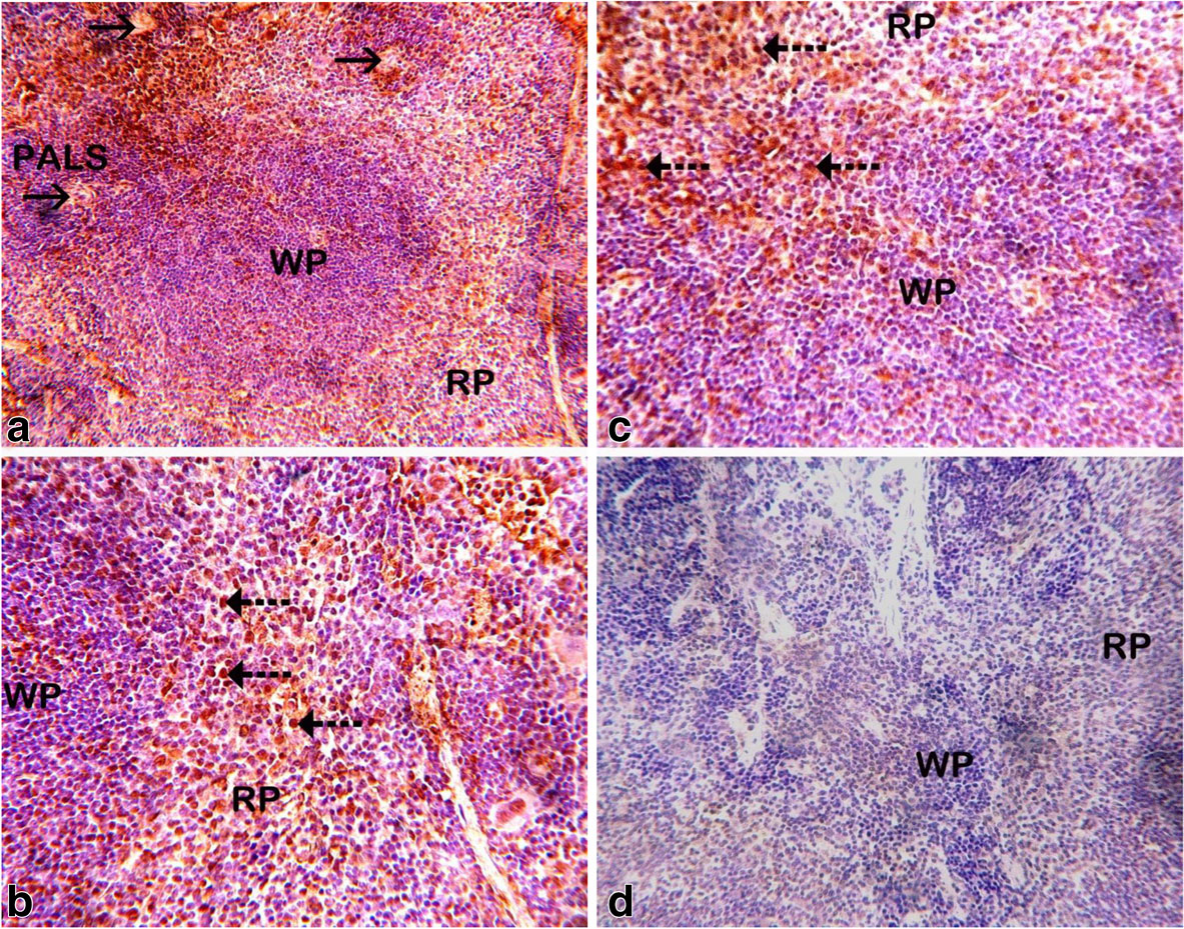
Immunohistochemical images of the spleen section, from mice treated with TME at the rate of 1500 mg/kg, 8 hourly intervals for three days consecutively. In the white pulp, PALS region of the spleen surrounding central artery (*black* arrows) appear darker as it is predominantly occupied by small lymphocyte in (**a**). In (**b** and **c**), detection of ssDNA in apoptotic cells, a marker for apoptosis is shown as a *brown reaction* due to DAB peroxidase reaction (*dotted line arrows*). The reaction is severe in the *red pulp* and areas on the marginal zone of the* white pulp*, some of the reactions are also seen in the PALS and the lymphoid follicles. Image D is from mice treated with PBSG at 0.1 ml/ 10 g as the extract at 8 hourly for three days consecutive for comparison. Magnifications, (**a**) 100 x; (**b**, **c** and **d**) 400 x

**Fig. 12:**
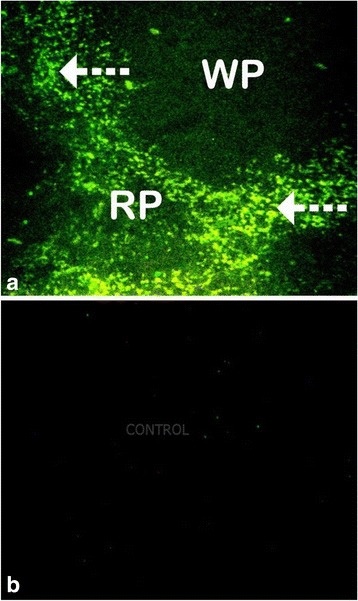
Immunohistochemical images of the spleen section, from mice treated with TME at the rate of 1500 mg/kg, 8 hourly intervals for three days consecutively. Immunoreactivity labeling indicative of apoptotic cells (*white arrows*) largely seen at the periphery of the *white pulp* and extends in the *red pulp* in (**a**). Some cells positive for ssDNA immunoreactivity are also seen in the white pulp in (**b**). Magnifications, (**a** & **b**) 400×

Detection of immunoreactivity labeling of ssDNA, a marker of apoptotic cells was consistently observed from spleen sections of mice treated with SE (G11) and TME (G12) by both DAB peroxidase and secondary antibody (Alexa Fluor® 488-conjugated goat anti-rabbit IgG). The ssDNA was shown as a brown reaction resulting from DAB peroxidase reaction (Fig. 11 a, b and c). The reaction was shown to be severe in the red pulp and areas on the marginal zone of the white pulp, some of the reactions were also seen in the PALS and the lymphoid follicles (B and C). The PALS region of the white pulp of the spleen surrounding the central artery (black arrows) appeared darker as it is predominantly occupied by small lymphocytes in (A). However, from mice treated with PBSG at 0.1 ml/ 10 g as the extract at 8 hourly intervals for 3 days consecutive did not show any reaction (D). On one hand, severe apoptosis of lymphocytes was also confirmed by the secondary antibody as observed in the immunoflorescent images of the spleen in (Fig. 12). Immunoreactivity labeling indicative of apoptotic cells (white arrows) was largely seen at the periphery of the white pulp and extended in the red pulp (A). In PBSG treated mice, only few cells positive for ssDNA immunoreactivity were observed in the white pulp in (B).

### Chemical composition of methanolic extract

**Fig. 13:**
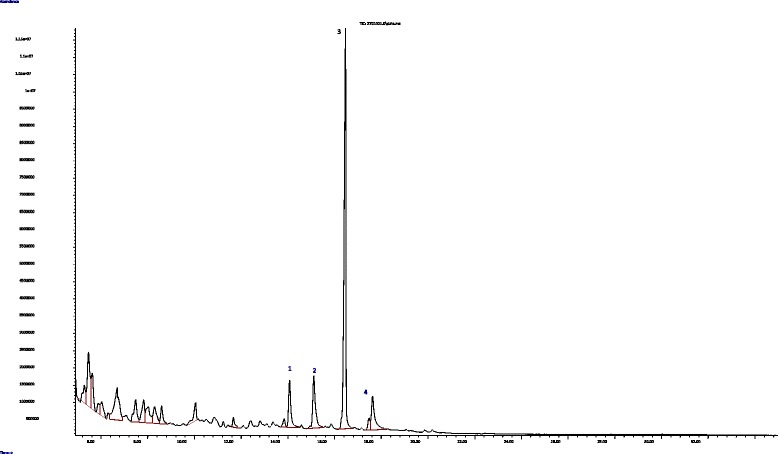
GC-MS Chromatogram of *Commiphora swynnertonii* methanolic stem bark extract. The labeled number represents: (1) Hexadecane-1,2-diol, (2) 9-Eicosene, (E)-, (3) Borneol, (4) Dichloroacetic acid, tridecyl ester

The presence of the components in *C. swynnertonii* methanolic stem bark is presented in Table 4 and Fig. 13.

**Table 4:** Major phytochemical components from *Commiphora swynnertonii* methanolic stem bark extract identified by GC-MS

Peak	Component	RT	% Composition
1	Undecanoic acid	5.287	2.269
2	.beta.-D-Glucopyranose, 1,6-anhydro-	5.462	7.229
3	1,4-Benzenediol, 2-methoxy-	5.625	4.004
5	Methyl-.alpha.-d-ribofuranoside	6.028	2.219
6	2H–Pyran-2-one, tetrahydro-4-hydroxy-6-pentyl-	6.689	8.084
7	Heptanoic acid	7.481	3.512
8	Benzoic acid, 4-hydroxy-3-methoxy-	7.829	3.190
9	endo-Borneol	8.018	3.415
10	alpha.-Santoline alcohol	8.292	2.911
11	Phenol, 3,4,5-trimethoxy-	8.595	2.070
15	Hexadecane-1,2-diol	14.076	5.405
16	9-Eicosene, (E)-	15.114	7.311
17	Borneol	16.466	35.852
19	Dichloroacetic acid, tridecyl ester	17.630	5.780

*RT* retention time

## Discussion

This study has evaluated the in vivo effect of ethanolic stem bark extract of *C. swyynertonii* on *T. congolense* and selected immunological components in a mouse model. There was considerable reduction in the levels of parasitaemia in the mice treated with the extract at 8 hourly intervals. The significant suppression of *T. congolense* at 2000 mg/kg a day post therapy and at 1000 mg/kg on day 2, 3 and 4 is an indication that the ethanolic stem bark extract of *C. swynnertonii* possesses antitrypanosomal activity. Similar observations using extracts from different plants have been observed by other workers [29, 30]. Therefore, the current results confirm our earlier report which had similar results [21]. In addition, the study shows that it was necessary to administer higher dose of the extract which was however administered three times per day at 8 hourly intervals to attain the observed effect. The better result following treatment at 8 hourly intervals could be affected by the short half-life of extract constituents thus being unable to persist long enough to exert a pronounced effect on the parasites [31]. Also, enzymatic inactivation of the active compounds and impaired absorption from the site of administration might lead to insufficient concentration and duration for any therapeutic effect at the target organs [32].

On the other hand, a dose of 2000 mg/kg failed to maintain its suppressive activity against *T. congolense* during therapy. High concentrations of all extracts of *C. swynnertonii* except the leaf extract have been suggested to acutely affect the biological systems [20]. Nevertheless, a dose of 1000 mg/kg provided an increase in therapeutic effect up until the end of therapy. Thereafter, levels of parasitaemia started to rise following cessation of therapy. This could be attributed to cumulative effect and tolerance by the mice. The extract could possess trypanocidal activity, cessation of therapy resulted in termination of trypanocidal effect of residual concentration. *T. congolense* has been found to localize the microvasculature of organs such as brain, heart and skeletal muscles [33, 34]. Hence, re-appearance of parasitaemia in mice that showed complete clearance may be attributed to residual parasites from these hiding sites. Therefore, other factors such as antibody response are involved in facilitating total clearance of trypanosomes infection [35]. The extract contains many phytochemical compounds such as those presented in this study [20], the observed effect may be accounted by several possible mechanisms working separately or in concert [36, 37]. Diminazene diacteurate at 3.5 mg/kg cleared the trypanosomes in blood on third day post therapy though few mice relapsed on 17^th^ day. This might probably be due to administered dose being subcurative in mice [38, 39].

The extracts induced a slight increase but an overall insignificant total white blood cell count in mice. However, TME at 500 mg/kg and 1500 mg/kg down modulated the percentage of lymphocytes whilst those of neutrophils and monocytes were up modulated. This finding differs from Bakari and colleagues [40] who reported a significant up modulation of monocytes and lymphocytes in chicken. The difference might be attributed to species variations, mode of administration, the difference in dosage and the type of extract employed. Nevertheless, SE at 1500 mg/kg decreased the percentage of lymphocytes while the percentages of other leukocytes were insignificantly affected. This suggests that SE exhibits a relatively less effect on the immune system than TME. One of the evidence is the marked reduction in size of lymphoid follicles and depletion of the marginal zone, one of the changes imposed by immunomodulatory agent [41].

On the other hand, the mice that were not infected but treated with the extracts had mild to marked histological changes in spleen. At a low dose of both TME and SE, histological changes were indistinguishable from the control. A higher dose of SE induced apoptosis and white foci of variable sizes in the white pulp that extended in the red pulp. Such histological changes are clearly different from the one induced by the higher dose of TME which includes: a marked reduction of the size of white and red pulp compartments, the PALS had normal cellularity and size, lymphoid follicles and the marginal zone were almost depleted. This may provide an indication that TME at the higher dose could possess more toxic effect than the SE. One such evidence were observed in other organs from the mouse that received 2000 mg/kg, 8 hourly intervals but died on 2^nd^ day of therapy. A marked cytoplasmic vacuolation of hepatocytes, some pyknotic hepatocytes, Küpffer cell hyperplasia and a mild hydropic degeneration of cortical-tubular epithelium and glomerulus were the characteristic features in the liver and kidney respectively.

Previous studies have stipulated that changes in the size and density of the PALS and or marginal zone, and a change in lymphoid follicles of the spleen is associated with exposure to immunomodulatory agent [42, 43]. It is clearly documented that apoptosis in the splenic white pulp as observed in this study at the higher dose of SE and TME is a typical feature of compounds that induce lymphocyte toxicity [44]. Such feature is coupled with a decrease in peripheral lymphocytes and down regulation of humoral mediated immunity [45]. Observations by Bakari [20] showed that antibody titre was inversely proportional to dosage of resinous extract of *C. swynnertonii*. Nevertheless, the depletion of lymphoid follicles and marginal zone at higher dose of TME and the mild decrease at the low dose could be associated with deficit in T cell independent immune response [42, 46] which is essential in humoral response [47]. Thus, in the light of our findings, it is hereby hypothesized that, failure of crude extract of *C. swynnertonii* to clear the infection of *T. congolense* is attributed to its suppressive effect on humoral mediated immunity. To this effect, there is a sufficient amount of evidence to speculate that, besides its direct effect on the trypanosomes, the ethanolic stem bark extract of *C. swynnertonii* activated the cell mediated immune response thus down modulated the levels of parasitaemia but failed to clear the parasites due to its negative effect on humoral mediated immunity.

The role of immunity in facilitating clearance of trypanosome infection in host is well documented [35, 48, 49]. Considering trypanosome’s ability of evading the immunity in favor of its survival in a host, switching to a different variant antigenic type (VAT) to avoid antibody mediated destruction [50, 51], It is less successful to attain therapeutic effect through administering crude extract of *C. swynnertonii*. However, our study indicates that ethanolic extract of *C. swynnertonii* could be a potential source of antitrypanosomal compound(s).

## Conclusion

This study has provided evidence that even at its crude state, ethanolic extract of stem bark of *C. swynnertonii* possesses in vivo trypanocidal activity. Although the activity is probably impaired by its negative effect on the humoral mediated immune response, the extract of *C. swynnertonii* could still be a potential source of antitrypanosomal compound(s). It is suggested that fractionation of the phytochemical compounds to isolate the ones possessing trypanocidal activity could minimize the undesirable effect. Further studies are recommended to determine the potential of stem bark extract of *C. swynnertonii* as an alternative source of lead compound(s) for trypanocidal drug discovery.

## Abbreviations

hr.: Hour
Min: Minutes
mm: Millimeter
PBS: Phosphate buffered saline
PBSG: Phosphate buffered saline with glucose
